# Evaluation of the Effect of Garlic Tablet as a Complementary Treatment for Patients with Diabetic Retinopathy

**DOI:** 10.1155/2022/6620661

**Published:** 2022-07-14

**Authors:** Mehrdad Afarid, Elham Sadeghi, Mohammadkarim Johari, Ehsan Namvar, Fatemeh Sanie-Jahromi

**Affiliations:** Poostchi Ophthalmology Research Center, Department of Ophthalmology, School of Medicine, Shiraz University of Medical Sciences, Shiraz, Iran

## Abstract

**Purpose:**

The aim of this study was to investigate the effectiveness of garlic (Allium sativum L.) tablets as a complimentary herbal medication in diabetic macular edema.

**Methods:**

A total of 91 diabetic participants (117 eyes) with central involved macular edema underwent a double-blind randomized trial. The patients used garlic tablets (500 mg) (2 tab/day) or placebo for 4 weeks and subsequently were examined by an expert ophthalmologist. Clinical manifestations including the best-corrected visual acuity (BCVA, logMAR), central macular thickness (CMT, *μ*m), and intraocular pressure (IOP) were measured as the main outcomes.

**Results:**

BCVA was significantly improved by a 0.18 decrease in mean logMAR value in the garlic-treated patients in comparison with 0.06 in the control ones (*P* value = 0.027). CMT was decreased in both groups by a 102.99 *μ*m decrease in the garlic group compared to 52.67 *μ*m in the placebo group, albeit in a nonsignificant manner (*P* value: 0.094). IOP was decreased in the garlic group by 1.03 mmHg (*P* value: 0.024) and increased by 0.3 mmHg (*P* value: 0.468) in the placebo group.

**Conclusion:**

Our trial suggests that garlic supplements can improve visual acuity, decrease the CMT and lower the IOP, and can be considered as an adjuvant treatment in patients with diabetic macular edema. Garlic was satisfactorily tolerated in diabetic patients, and no significant adverse effect interrupting the safety profile was observed.

## 1. Introduction

Diabetic retinopathy (DR) is a common eye disease in adults that can lead to blindness, contributing to retinal neurodegeneration and microvascular abnormalities. DR is often associated with physical and psychological complications such as decreased quality of life and increased psychosocial problems [1, 2]. Fundus examination revealed microaneurysms, vascular leakage, exude, and capillary occlusion, with fibrous and neovascular proliferation [3]. Intravitreal antivascular endothelial growth factor (anti-VEGF), Laser photocoagulation, vitreoretinal surgery, and triamcinolone injection are the current treatments [4].

The results of several large clinical studies proposed that control of blood sugar and blood pressure plays an important role in preventing the progression of DR [5, 6]. Despite the emergence of new therapeutic strategies for diabetic retinopathy, many of these drugs have not been effective in controlled randomized trials [7]. Therefore, there is a need to develop new drugs and approaches to manage DR. Given the long history of herbal medicines in the management of diabetes mellitus and its complications [6], it seems that by examining some herbs in clinical trials, credible evidence-based results can be released indicating the efficacy of herbal medication as a complementary treatment to inhibit DR complication or postpone the disease progression.

Garlic (Allium sativum L.) is a traditional drug-containing diverse bioactive compound, including sulfur-containing compounds such as alline and allicin, saponins, and phenolic contents. Several biological effects have been reported for garlic including anti-inflammatory, antioxidant, antidiabetic, immunomodulatory, antiatherosclerotic, antihypertensive, and cardioprotective effects [8]. Garlic has also been shown to exert antioxidants and neuroprotective effects, inhibit retinal ischemia, and decrease the expression of VEGF, hypoxia-inducible factor 1 alpha (HIF-1*α*), inducible nitric oxide synthase (iNOS), and metalloproteinase (MMP)-9 [9–11].

To date, no report has been published on the efficacy of garlic supplementation to treat diabetic retinopathy. Since the effect of garlic as an antidiabetic and anti-VEGF agent has been confirmed in previous studies, for the first time, we used garlic as a complementary treatment for DR patients in this project and evaluated the effectiveness of this herbal treatment in the control of DR and diabetic macular edema (DME).

## 2. Methods

### 2.1. Experimental Design

This study was conducted in the Shiraz University ophthalmology center in Shiraz, Iran, between 2020 and 2021, and received the ethical approval of Shiraz University of Medical Sciences (IR.SUMS.MED.REC.1399.052). The trial also was registered in the Iranian Registry of Clinical Trials (IRCT20200415047083N1). The required total number of participants (case vs. control) was calculated with the help of statisticians and related studies. The trial was designed as a double-blind randomized trial.

### 2.2. Contributors

Spectral-domain optical coherence tomography (SD-OCT) (Spectralis, Heidelberg, Germany) was done for diabetic participants, and patients with a central macular thickness (CMT) of more than 350 *μ*m were included in the study. The follow-up was regularly done in this university eye center, and standard treatments were performed for them (according to http://www.drcr.net/, Diabetic Retinopathy Clinical Research network protocols). The study was supervised by an expert vitreoretinal surgeon. It should be noted that the surgeon was not informed of the randomization pattern of participants.

Patients with monocularity, glaucoma, previous retinal vascular occlusion, epiretinal membrane or vitreomacular traction on fundus examination or OCT, significant cataract, posterior capsular opacification, or vitreous hemorrhage that prevents posterior fundus view, previous history of vitrectomy surgery, previous history of cataract surgery during last three months, high myopia, getting a systemic or intravitreal injection of immunosuppressive and corticosteroid drugs, getting warfarin, clopidogrel or any blood thinner drugs, history of radiotherapy, cancers, gastrointestinal disease, and liver or kidney failure were excluded from the study. The study was performed on one or both eyes considering the aforementioned inclusion and exclusion criteria.

### 2.3. Randomization and Interventions

In the first pilot study, fourteen cases were randomized by the random digits table in equal proportions to oral treatment with a garlic tablet or a placebo tablet. The standard dose of the oral garlic tablet was estimated based on its safety studies in hypertension and other cardiovascular risk factors treatment [12, 13]. Each coated garlic tablet contains 500 mg garlic (Allium sativum) granulated powder and stands on 2-3 mg allicin (Garcin 500; Goldaru Co, Isfahan, Iran). The cases were used two tablets a day with lunch for four weeks, and they were visited weekly. Placebo tablets contain 60% lactose, 35% avisol, 4.5% cornstarch, and 0.5% magnesium stearate, packaged in a package similar to garlic tablets. The placebo was administered similarly to garlic pills.

At baseline, intraocular pressure (IOP) (air-puff tonometry by TOPCON KR 800), best-corrected visual acuity (BCVA) (Snellen chart), refraction (auto-refractometer by ZIESS Visuref 100), slit-lamp biomicroscopy, and fundoscopic examination were assessed by an unrelated ophthalmoscope, and CMT was evaluated by SD-OCT. After the fourth week, all these items were repeated after the initiation of the tablets. For BCVA measurement, the value of the logarithm of the minimum angle of resolution (logMAR) scale was used. Eye examination was performed randomly. The randomization of subjects was done by an assistant who had no role in treating the patients.

According to the pilot study results, after confirming no significant general or ocular complications, the sample size was broadened to 91 cases. All the cases received their treatments of DME by intravitreal injection of bevacizumab (1.25 mg/0.05 ml of Avastin, AryoGen, Iran) and laser photocoagulation and received oral garlic or placebo tablet as a complementary treatment. Due to randomization, we minimized the effect of previous treatments in creating any impact on the results.

### 2.4. Clinical Outcomes

Changing logMAR and the CMT (*μ*m) were measured as a primary outcomes, and changing IOP and any general or ocular complication were secondary outcomes. Since garlic is a blood thinner, significant ocular complications such as new hyphema and vitreous or retinal hemorrhage were determined.

### 2.5. Statistical Method

In this study, the relative frequency was applied to report the qualitative variables, and for quantitative variables, mean and standard deviation (SD) was reported. Mann–Whitney test was accomplished for analyses of the treated patients vs. control. The statistical significance level of the test was considered as *P* value <0.05. The SPSS version 23 (SPSS, Inc., Chicago, IL) was used for data management and statistical analysis.

## 3. Results

### 3.1. Baseline Patients' Demographics

**Figure 1:**
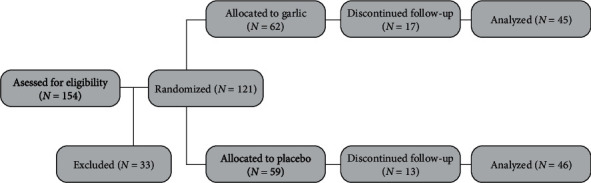
Consort flow diagram.

A total of 154 patients receiving treatment for CI-DME were studied. One hundred twenty-one of them were included due to inclusion criteria. Eight patients discontinued the study in the garlic group because of difficulty adhering to follow-up due to COVID-19 quarantine. Eight of them garlic were removed from this study due to not taking medicine according to the instructions. One patient in the garlic group manifested gastrointestinal upset on the first administration day and terminated the treatment. Seven patients left the study in the placebo group because of the farther distance, and six patients discontinued the study due to COVID-19 quarantine.  Figure 1 shows the CONSORT flow chart representing the flow of participants in the study.

Of the 91 cases of central involving macular edema in the sitting of diabetic retinopathy, 45 and 46 patients were randomly divided into two groups representing the cases and the controls, respectively. Participants had a mean age of 61.91 ± 6.35 years in the case group and 60.50 ± 7.04 years in the control group (*P* value: 0.319). A significant number of participants in this study were female in both the case and control groups (83.1% vs. 56.9%, respectively), indicating a significant difference (*P* value: 0.002).

Baseline laboratory evaluation revealed that the mean of fasting blood sugar (FBS) in the case vs. control group was 175.06 ± 73.88 vs. 177.5 ± 62.73 mg/dL, indicating no remarkable alteration (*P* value: 0.487). HbA1c analysis also showed no significant change in the case/control treatments (8.63 ± 2.15% vs. 8.37 ± 1.28%, *P* value: 0.492). Demographic and laboratory data are shown in Table 1. Based on the inclusion and exclusion criteria, 117 eyes from all cases were included (59 eyes in the case group and 58 in the control group). Baseline fundus examination revealed that in the case group, 13.6% of cases were severe NPDR, 11.9% PDR, and 74.6% were regressed PDR, and in the control group, 20.7% were severe NPDR, 6.9% were PDR, and 72.4% were regressed PDR. Statistical analysis confirmed that no significant difference was detected between the two groups in type of diabetic retinopathy (*P* value: 0.437, Table 2).

In 4 weeks of follow-up, an anti-VEGF injection was performed for all the cases in both groups. Eleven patients (six in the case and five in the control group) received pan-retinal photocoagulation (PRP) treatment.

### 3.2. BCVA, CMT, and IOP

Baseline means BCVA LogMAR in the case group were 0.93 ± 0.36 (20/160 Snellen chart), and in the control group were 0.85 ± 0.33 (20.125 Snellen charts). After a four-week follow-up, the garlic receiving patients showed a more significant improvement in BCVA in comparison with control ones (0.18 decrease mean logMAR value in garlic treatment compared to 0.06 in the placebo treatment, Mann–Whitney test for log Mar *P* value =0.027) (Table 3). The baseline means of the CMT in the case group were 513.66 ± 120.79 *μ*m, and in the control group were 501.39 ± 133.42 *μ*m. Four weeks after the treatment, patients taking garlic had a more decrease in CMT compared with the placebo group (102.99 *μ*m decrease in the garlic group compared to 52.67 *μ*m in the placebo group); however, this decrease was not statistically significant (independent *t* test *P* value: 0.094) (Table 4).

The baseline means of IOP in the case group were 15.40 ± 3.68 mmHg, and in the control were 15.51 ± 2.74 mmHg. In the fourth week, the mean IOP was decreased in the garlic group by 1.03 mmHg (*P* value: 0.024) and increased by 0.3 mmHg (*P* value: 0.468) in the placebo group. Independent *t*-test showed that the IOP lowering effect of garlic compared to placebo was statistically significant in the four-week follow-up (*P* value =0.036) (Table 5).

### 3.3. Safety

No significant ocular or systemic complications were seen in the cases except a mild gastrointestinal upset in one of the patients receiving garlic; notably, this side effect which was self-limited and predominantly resolved by the garlic tablets discontinuation. This patient was removed from the study.

## 4. Discussion

DR is one of the main causes of blind impairment and vision loss in the world [14]. The most important risk factors for the pathogenesis of the disease include the duration of diabetes, hyperglycemia, hypertension, and hyperlipidemia. The control of these factors seems to better lead us to target effective management of the disease [15]. The cellular and molecular changes in the process of retinopathy have recently been studied; several molecular and cellular pathways have been investigated, and according to these findings, novel therapeutic targets have been developed [16]. The main strategy for the management of diabetic retinopathy is retinal niche modification in terms of the availability of mediators, especially cytokines, inflammatory agents, and angiogenic factors [17, 18]. Intravitreal anti-VEGF injection and laser photocoagulation are among the common therapies for diabetic retinopathy. In addition to chemical treatments, herbal remedies have been used as adjunctive therapies for diabetic retinopathy [19]. Garlic is a traditional medicinal plant that has been reported to be useful in the control of diabetes, hypertension, hyperlipidemia, and cardiovascular diseases [20–22]. The experimental data has confirmed the regulatory effect of garlic on various cellular signaling pathways [23]. In vitro studies on 3T3-L1 adipocytes indicate that garlic suppresses LPS-induced inflammatory response by promoting anti-inflammatory gene expression. Moreover, garlic prevents the expression of inflammatory cytokines like IL-6 and MCP-1 [24]. The immunomodulatory effect of garlic extract has also been demonstrated in experimental studies, and it has been shown that garlic can control the secretion of TNF-*α* and nitric oxide (NO) by macrophages dose-dependently [25]. The antiangiogenic effect of garlic extract has been estimated in several in vitro studies. Recently, it has been shown that garlic could decrease the expression level of VEGF in A549 lung cancer cells, with no cytotoxic effect [26]. It has also been shown that the antiangiogenic effect of garlic compounds is due to its inhibitory effect on particular signaling pathways in nondiabetic cell models, including VEGF, AKT/ERK, and NO pathways [27]. Moreover, several reports are indicating the neuroprotective and neuro-regenerative effects of garlic [28–31]. Accordingly, garlic is suggested to enhance the antiangiogenic effect of intravitreal anti-VEGF agents, protect the neural retina from oxidant damages, suppress the inflammation process that is responsible for the pathogenesis of DME, and can be an effective complementary treatment in the management of diabetic retinopathy.

In the present study, diabetic retinopathy patients were supplemented with garlic, and the main clinical manifestations of patients in the pre- and post-garlic consumption period were examined. Our study implied that short-term garlic tablet consumption is effective in visual acuity improvement and reducing CMT when it is used as an adjuvant treatment with anti-VEGF agents in the management of DME. It was also demonstrated that garlic might be effective in lowering IOP. A dosage of two tablets daily (1 gr of garlic granulated powder containing 4-6 mg allicin S-allyl cysteine) significantly increased BCVA compared to a placebo over four weeks; although it clinically decreased the CMT in our patients, this value was not statistically significant.

DME, the leading cause of vision loss in diabetic retinopathy, is induced by the accumulation of extracellular fluid in the retina that disrupts the retinal cell structure [32]. The results of this study showed that garlic decreased the macular thickness after 4 weeks of consumption; however, this difference was not statistically significant. This outcome might be due to the short follow-up period. Koushki M et al.'s meta-analysis study suggested that garlic can be applied as a complementary medication for the management of metabolic diseases. Subgroup analysis showed that the inflammatory markers such as IL-6, TNF-*α*, and CRP were markedly decreased in subgroups of >8, >6, and ≥4 weeks of treatment periods, respectively, and hence, the effectiveness of garlic supplementation is directly related to the duration of consumption [33]. This data might explain why the amount of CMT decrease was not statistically significant in our study.

We also showed that the patients who received garlic supplements manifest a significant improvement in visual acuity. This observation might be interpreted by the effect of garlic on the ocular perfusion flow. Garlic intake is demonstrated to have a remarkable role in increasing blood flow [34]. Recently it has been shown that the ocular blood flow of diabetic retinopathy patients is significantly decreased due to the wall thickness of their retinal vessels [35]. On the other hand, the increase in optic nerve blood flow is reported to have a pivotal role in the improvement of visual acuity in glaucoma patients [36]. Garlic is also demonstrated to ameliorate the process of neurodegeneration of the neural retina in animal models [37], which might support our finding. Given that ocular ischemia due to vascular endothelium damage is the main cause of diabetic retinopathy and damage to the neural retina, it is suggested that garlic intake might improve visual acuity by increasing the ocular blood flow and protecting the neural retina from oxidant stress. However, more prolonged studies or higher doses of garlic may show better effects on ocular blood profile.

Our data also showed that garlic intake could remarkably decrease the IOP. This study is in line with previous reports on the effect of garlic compounds on IOP [38]. Garlic can reduce IOP by contracting the ciliary muscles and assisting in the outflow of aqueous humour [39]. Notably, it has been shown that multiple injections of anti-VEGF might increase the IOP [40]. The present study demonstrated that garlic consumption might be helpful to suppress IOP increase after anti-VEGF treatment.

A review of the recent studies confirms that complementary therapies have recently found an important place in the treatment of diabetic retinopathy [41]. Garlic is featured by several biological properties including antioxidant effects [42], and today, combined antioxidant therapy (CAT) has gained popularity, as shown by different studies [43].

In addition, Lafuente et al.'s study represented successful outcomes for adding a combined antioxidant treatment to anti-VEGF in the treatment of DME [44, 45]. These studies confirmed the results of our study. Accordingly, garlic can be used as an adjunctive treatment to anti-VEGF for DME treatment.

It should be noted that garlic has anticoagulant and antihypertensive properties and may increase the tendency to bleed in susceptible patients. Therefore, it should be prescribed with caution in these patients. Although the present study confirmed that garlic consumption was safe and no adverse event was observed in patients who received garlic pills, no significant vitreous hemorrhage or other organ bleeding was observed in our study. We also checked the bleeding time and excluded the patients with a history of active gastrointestinal (GI) ulcers or cerebral vascular accidents (CVA).

The main limitation of our study was its duration. Additionally, some circumstances, mainly the COVID-19 pandemic, caused poor follow-up of patients, and we could not follow patients for longer periods. The other restrictions and confounding factors were common dietary habits. In our country, garlic is used as a flavoring that may influence the results of the study. We tried to decrease the effect of these factors by randomization. For future research, the use of natural products such as garlic products to help treat diabetics, especially retinopathy, is recommended in studies with larger sample sizes and the control of confounding factors such as diet and other risk factors. The possible effects of long-term use of garlic products on reducing the incidence of vascular disease can also be examined. The use of higher doses of the drug and drug interactions with other drugs commonly used by diabetics are topics that may be the subject of future research.

## 5. Conclusion

Overall, the presented data indicate that garlic supplementation can enhance visual acuity and decrease IOP in patients with diabetic retinopathy. However, we could not detect a significant change in macular thickness between garlic-treated patients in comparison to the control group. In addition to many experimental and preclinical studies reporting the anti-inflammatory, antiangiogenic, antioxidant, and neuroprotective effects of garlic, the present study, for the first time, provided valuable evidence on the improving effect of garlic consumption on diabetic retinopathy patients. Further clinical investigations are required to better evaluate this product. The ease of access, ease of use, and natural identity of garlic are its positive aspects. Garlic application as a complementary drug in patients with diabetic retinopathy might reduce the need for intravitreal injection or retinal surgery to improve visual acuity and control IOP in patients. This finding might extend novel platforms to the management of diabetic retinopathy.

## Figures and Tables

**Table 1:** Baseline patients' characteristics.

Characteristics	Garlic (*N* = 45)	Placebo (*N* = 46)	*P* value
Mean age, years (SD)	61.91 ± 6.35	60.50 ± 7.04	0.319
Age range, years	43-76	42-79	
Female (%)	83.1%	56.9%	≤0.001
FBS (mg/dL ± SD)	175.06 ± 73.88	177.5 ± 62.73	0.487
HbA1C (± SD)	8.63 ± 2.15%	8.37 ± 1.28	0.492

FBS: fasting blood sugar; HbA1C: glycated hemoglobin.

**Table 2:** Baseline fundus ophthalmoscopy.

Characteristics	Garlic (*N* = 58)	Placebo (*N* = 59)
Severe NPDR, *n* (%)	13.6%	20.7%
PDR, *n* (%)	11.9%	6.9%
Regress PDR, *n* (%)	74.6%	72.4%

**Table 3:** Best corrected visual acuity in LogMAR of the two groups on baseline and fourth weeks.

LogMAR visual acuity	Garlic (*N* = 59)	Placebo (*N* = 58)	Minimum garlic	Minimum placebo	Maximum garlic	Maximum placebo
The baseline value of BCVA (± SD)	0.93 ± 0.36	0.85 ± 0.33	0.20	0.20	1.60	1.60
After the 4th week value of BCVA (± SD)	0.75 ± 0.36	0.79 ± 0.36	0.20	0.10	1.60	1.60
Change	-0.18	-0.06	0.00	-0.10	0.00	0.00
*P* value	≤0.001	0.014				

**Table 4:** Central macular thickness (CMT, *μ*m) of the case and control groups during the pre- and post-treatment period.

CMT	Garlic (*N* = 59)	Placebo (*N* = 58)	Minimum garlic	Minimum placebo	Maximum garlic	Maximum placebo
The baseline value of CMT (± SD)	513.66 ± 120.79	501.39 ± 133.42	365	352	838	904
After the 4th week value of CMT (± SD)	410.67 ± 155.47	448.72 ± 141.65	177	186	874	813
Change	-103	-53	-188	-166	+36	-91
*P* value	≤0.001	0.005				

**Table 5:** Intraocular pressure (IOP, mmHg) of the case and control groups during the pre- and post-treatment period.

IOP	Garlic (*N* = 59)	Placebo (*N* = 58)	Minimum garlic	Minimum placebo	Maximum garlic	Maximum placebo
The baseline value of IOP (± SD)	15.40 ± 3.68	15.51 ± 2.74	10	10	30	22
After the 4th-week value ± SD (IOP)	14.37 ± 2.82	15.81 ± 2.48	10	11	24	21
Change	-1.03	+0.03	0	+1	-6	-1
Value	0.017	0.532				

## Data Availability

The data will be available on request.
